# Editorial: Rising stars in Evolutionary and Genomic Microbiology: 2022

**DOI:** 10.3389/fmicb.2022.1098065

**Published:** 2022-12-16

**Authors:** Alla Lapidus, Ernesto Perez-Rueda

**Affiliations:** ^1^Center for Algorithmic Biotechnology, St. Petersburg State University, Saint Petersburg, Russia; ^2^Unidad Académica Yucatán, Instituto de Investigaciones en Matemáticas Aplicadas y en Sistemas, Universidad Nacional Autónoma de México, Mérida, Mexico

**Keywords:** microbiology, genomics, antibiotic resistance, metatranscriptomic, metagenomics, CRISPR

## Introduction

Recognizing the future leaders of Evolutionary and Genomic Microbiology is fundamental to safeguarding tomorrow's driving force in innovation. This Research Topic will showcase the high-quality work of internationally recognized researchers in the early stages of their careers. We aimed to highlight research by leading scientists of the future across the entire breadth of Evolutionary and Genomic Microbiology, and present advances in theory, experiment and methodology with applications to compelling problems.

Among the chromosomal structures and genomic differentiation articles,Wang et al. leading a group in the Beijing Institute of Biotechnology in China investigated changes in chromosomal structure of Klebsiella Pneumoniae under Simulated Microgravity (SMG). The authors, asked whether phenotypic changes induced by SMG in the subpopulations of *K. pneumoniae* are driven by genome higher-order structural changes. By the combination of genome-wide chromosome conformation capture (Hi-C), RNA-seq and whole-genome methylation (WGS) analyses, they found that the along with the global chromosome interactions change, the compacting extent of M1, M2 subpopulations were much looser under SMG and even with an increase in active, open chromosome regions. In addition, transcriptome data showed that the function of most differentially expressed genes (DEGs) were mainly associated with membrane fractions. Additionally, WGS analysis revealed that methylation levels were lower in M1 and M2. Finally, they discovered that most upregulated DEGs were enriched in the boundary regions of the variable chromosomal interaction domains (CIDs), in which genes regulating biofilm formation were mainly located. These results suggest that K. pneumoniae may regulate gene expression patterns through DNA methylation and changes in genome structure, thus resulting in new phenotypes in response to altered gravity.

In contrast, the group of Stanojković et al. from the University in Prague, Czechia explored the genomic differentiation and gene flow within the genus Laspinema (cyanobacteria). In this regard, the authors used phylogenomics and a population genomic approach to investigate the extent of local genomic diversity and the mechanisms underlying sympatric speciation of *Laspinema thermale*. They found two highly genetically diverse cryptic lineages of *Laspinema* with recombination occurring more frequently within than between them. That suggests the existence of a barrier to gene flow, which further maintains divergence.

As a consequence of the antibiotic resistances identified in the bacterial world as one of the greatest human and clinical challenges associated with different pathogenic organisms, from the Inner Mongolia Medical University, in China, Han et al. evaluated the epidemiological characteristics and molecular evolution mechanisms of carbapenem-resistant hypervirulent *Klebsiella pneumoniae* (hvKP). In particular, the authors summarize the evolutionary mechanisms of resistance and plasmid-borne virulence as well as the prevalence of CR-hvKP. Alternatively, Guillén-Chable et al. from the Universidad Nacional Autónoma de México, Mexico, described the presence and possible differences in the microbial resistome of four sites from the Yucatan coast through the evaluation of the composition and abundance of Antibiotic Resistance Genes (ARGs) using a high-throughput analysis of metatranscriptomic sequences. They found ARGs, which participate in the resistance to tetracycline, macrolide, rifamycin, fluoroquinolone, phenicol, aminoglycoside, cephalosporin, and other antibiotics. This study contributes to generating information about the current state of antibiotic resistance on the Yucatan coasts and probably, to other coastal sites.

User-friendly pipelines for *de novo* assembly of complex data like metatranscriptomic and metagenomic data are currently of a high demand. The group headed by Prjibelski, St. Petersburg State University, Russia describes “MetaGT: A pipeline for *de novo* assembly of metatranscriptomes with the aid of metagenomic data.” This pipeline is based on the idea of simultaneous usage of both metatranscriptomic and metagenomic sequencing data obtained from the same sample. MetaGT assembles metatranscriptomic contigs and fills in missing regions based on their alignments to metagenome assembly. It was demonstrated that metagenomic data greatly improve completeness of assembled transcripts compared to sequences assembled solely from metatranscriptomic data. As a result, presented approach allows to overcome known complexities and obtain complete RNA sequences, as well as estimate their abundances.

Using comparative genomics, Xu et al. in the Chinese Academy of Agricultural Sciences, China, evaluated a potential biocontrol mechanism for two strains of *Lysobacter enzymogenes*. For this, the complete genomes of two strains of *L. enzymogenes* were obtained and characterized. One of them, CX03, has been shown to exhibit a broad spectrum of antagonistic activity against a variety of bacteria, while CX06 exhibits antagonistic activity against various fungi and oomycetes. Annotation of functional genes showed that strains CX03 and CX06 have many genes or gene clusters associated with the biosynthesis of various secondary metabolites, which may be responsible for different antagonistic activity against various plant pathogens.

In contrast, the Genome analysis of the candidate phylum MBNT15 bacterium from a boreal peatland predicted its respiratory versatility and dissimilatory iron metabolism. In this work, Begmatov et al., leading a group in the Research Center of Biotechnology of the Russian Academy of Sciences, Russia, assembled a near-complete genome of a member of MBNT15 from a boreal peatland metagenome and used genomic data to analyze the metabolic pathways of this bacterium and its ecological role. Comparative genome analysis of the MBNT15 phylum revealed that the vast majority of its members are capable of aerobic respiration and dissimilatory iron reduction and some species also can reduce sulfur and nitrogen compounds, but not sulfate. Based on phylogenetic and genomic analyses, the novel bacterium is proposed to be classified as *Candidatus Deferrimicrobium borealis*, within a candidate phylum Deferrimicrobiota.

Finally, in the Research Topic, Prakash and Kumar from the Indian Institute of Technology Guwahati in India, achieved a transcriptional analysis of CRISPR I-B arrays of *Leptospira interrogans* serovar Lai and its processing by Cas6. The authors predicted *in silico* database seven CRISPR arrays in L. interrogans serovar Lai positioned between the two independent cas-operons. Here, we present the redefined repeat-spacer boundaries of the CRISPR subtype I-B locus of serovar Lai. Using the reverse transcription-PCR (RT-PCR), the authors account that the seven CRISPR are transcriptionally active in serovar Lai.

We believe that the field of Evolutionary and Genomic Microbiology is of great importance and its scope of interest is expanding. This is evidenced by the variety of topics and the quality of the manuscripts included in this Research Topic. We also appreciate the interest and reception from the scientific community and hope that this Research Topic will interest researchers in the fields of genomics, microbiology, antimicrobial resistance, biomedicine and bioinformatics, among others.

## Author contributions

All authors listed have made a substantial, direct, and intellectual contribution to the work and approved it for publication.

